# Is There a Role for Postmastectomy Radiation Therapy in Ductal Carcinoma *In Situ*?

**DOI:** 10.1155/2012/423520

**Published:** 2012-06-13

**Authors:** Manjeet Chadha, Jason Portenoy, Susan K. Boolbol, Alyssa Gillego, Louis B. Harrison

**Affiliations:** Department of Radiation Oncology, Beth Israel Medical Center, 10 Union Square East, New York, NY 10003, USA

## Abstract

*Background*. DCIS treated by mastectomy ensures high local control rates. There is limited data on risk for relapse and lack of clear indication for adjuvant radiation therapy (RT). We report a retrospective review on a population of DCIS patients treated with mastectomy. The objective was to identify the overall incidence of relapse, risk factors for local recurrence, and accordingly for whom adjuvant postmastectomy RT may be considered. *Methods*. This is an IRB-approved retrospective study on a prospective breast cancer database. From 1997 to 2007, we identified 969 patients with diagnoses of DCIS, among them 211 breasts in 207 patients were treated with mastectomy and comprise the study group. *Results*. With a median followup of 55 months (4.6 years) the 10-year relapse-free survival is 97%. Two of 211 breasts (0.9%) treated with mastectomy developed a local-regional recurrence. Both the relapses were among patients defined as having <1 mm final mastectomy margin. *Conclusions*. The rare local relapse after mastectomy limits our ability to reliably identify risk factors for relapse. The consideration for postmastectomy RT should be based on an individualized risk evaluating surgical technique used, presence of BRCA mutation, grade and extent of tumor, and proximity of lesion to the margin of resection.

## 1. Introduction

With the widespread use of screening mammography, the incidence of DCIS in the USA has exponentially increased over the past 30 years. In 1983, 4800 cases were diagnosed, and in 2011 the incidence had increased to an estimated 57,650 cases [1]. Currently, in the USA approximately one quarter of breast cancers are noninvasive at diagnosis. The surgical treatment for ductal carcinoma *in situ* (DCIS) includes breast conserving surgery (BCS) with or without adjuvant radiation therapy and mastectomy. For an increasing proportion of patients with DCIS, breast conserving therapy is favored. Nevertheless, there are approximately 30% patients in the United States who undergo mastectomy for a variety of reasons that include an inability to obtain clear margins after multiple excisions, the presence of diffuse microcalcifications suspicious for malignancy, BRCA mutation carriers, history of collagen vascular disease, and personal preference [2–5]. 

Although no prospective randomized trials comparing mastectomy to breast conserving therapy have been completed, the mortality using either treatment is low and the 10-year survival exceeds 95%. Local recurrence following mastectomy is not a common event with estimated recurrence rates in range of 1-2% [5–7]. The aim of this study was to identify the overall incidence of relapse and risk factors for local recurrence, and accordingly for whom adjuvant treatment including postmastectomy radiation therapy may be considered. In addition, this paper includes a review of the literature with a focus on local regional relapse with respect to surgical margins after mastectomy. 

## 2. Methods and Materials

This is an IRB approved retrospective review of a prospectively maintained breast cancer database on patients treated at the Cancer Center at Beth Israel Medical Center and Roosevelt Hospital. Between 1997 and 2010, 969 patients with the diagnosis of DCIS were identified in the database. Among these 207 patients underwent mastectomy. Four patients had synchronous bilateral DCIS treated with bilateral mastectomy. Therefore, 211 breasts (in 207 patients) treated with mastectomy constitute the study group. Patients with new diagnosis of DCIS or concomitant DCIS and lobular carcinoma *in situ* (LCIS) were included. All patients with history of prior invasive and/or microinvasive breast cancer were excluded. All clinical and histopathology characteristics were obtained from the database, cross-referenced with patient charts, and pathology reports were studied.  Table 1 summarizes the clinical characteristics. The median age at diagnosis was 50 years (ranging from 25 years to 88 years). In the majority of patients, the cancer was mammographically detected. The surgical procedures included mastectomy alone in 39 (18.5%) cases and mastectomy with sentinel lymph node biopsy or axillary sampling in 172 (81.5%) cases. Nuclear grade 1 was noted in 22 patients, grade 2 in 97 patients, and grade 3 in 92 patients. Resection margins were defined as clinically close and positive when DCIS was noted ≤1 mm and was scored as negative in all other instances. Among the study group 88.6% (*n* = 187) had negative margins and 11.4% (*n* = 24) had close or positive margins. None of the patients received postmastectomy radiation therapy (PMRT). Hormonal therapy was variably used at the discretion of the treating oncologist. All patients were followed by the treating physicians at regular intervals. 

## 3. Results

**Figure 1:**
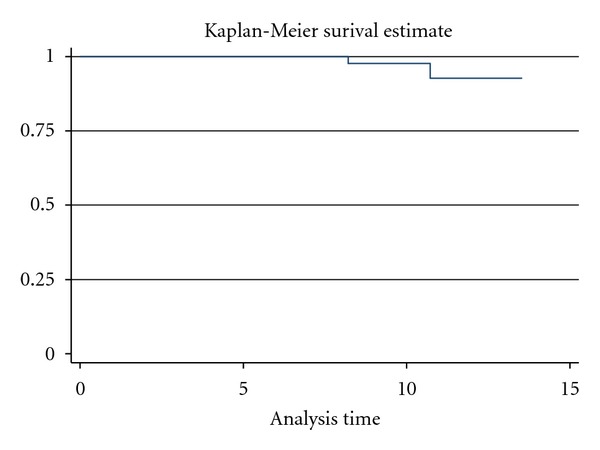
Local-Regional relapse-free survival curve for total cohort (*N* = 211).

The median followup is 55 months (4.6 years) (Figure 1). The 10-year local regional relapse-free survival is 97%. Among 211 patients, 2 patients developed a local-regional recurrence at 8.2 years and 10.7 years after initial diagnosis. The median time to local regional relapse was 9.5 years. In both instances, the recurrence had an invasive histology, and neither of these 2 patients had a skin sparing mastectomy. Both these failures were among the group of mastectomies reported to have clinical close/positive margins. On further analysis, comparing the 187 patients with negative margins and 24 patients with close/positive margin status, there was a significant correlation with risk for local regional relapse (*P* = 0.0125, Fisher's Exact Test). 

The clinical details of the 2 relapses are as follows.

**Relapse Case I**

The patient was diagnosed with mammographically detected DCIS at age 43. Following initial lumpectomy and reexcision that failed to yield clean margins, the patient underwent total mastectomy with sentinel lymph node biopsy. Final pathology reported intermediate-grade DCIS with associated comedo necrosis. Margin status confirmed DCIS present at the anterior medial-superior margin, and within one millimeter of the anterior margin at the lower inner quadrant, and at the medial-inferior margin. The sentinel lymph node was benign. She received no adjuvant therapies. In a 2007, a follow-up patient who underwent genetic testing was diagnosed to be a BRCA mutation carrier. She was without evidence of disease and underwent prophylactic oopherectomy and contralateral mastectomy at the time. In 2010, at a time interval of 8.2 years from initial diagnosis, she presented with a local recurrence at the superior lateral aspect of the reconstruction. At relapse, the patient underwent wide local excision and axillary lymph node dissection. Pathology confirmed DCIS and invasive recurrence in an area where presumably breast tissue was left behind. In addition, metastases to 7 out of 9 axillary nodes with extracapsular extension were reported. The recurrent tumor was ER and PR positive and Her2 negative. The patient received systemic chemotherapy, RT, and an aromatase inhibitor. Patient remains free of disease at last followup in January 2012. Remarkablly she also ran the breast cancer marathon in 2011.

**Relapse Case II**

Mammographically detected DCIS in a 52-year-old female. The patient underwent a total mastectomy with sentinel node sampling. Pathology noted intermediate-grade noncomedo DCIS present 1 millimeter from the superior margin. Three axillary lymph nodes were negative for metastases. Her follow-up history is significant for the diagnosis of an early-stage cervical cancer that was treated surgically. In 2010, at an interval of 10.7 years from initial diagnosis, the patient presented with ipsilateral supraclavicular metastasis. There was no evidence of metastatic disease on the ipsilateral chest wall, and the contralateral breast exam was clinically unremarkable. Pathology confirmed the recurrence to be consistent with breast primary and dissimilar from the cervix. The metastasis was ER and PR positive and Her2 negative. The patient was treated with Arimidex for 18 months and then switched to Faslodex. At the January 2012 followup, the patient remains alive with disease.

## 4. Discussion

In this retrospective series, very few patients had skin sparing mastectomy and majority of the patients had total mastectomy with lymph node sampling. Currently, however, skin sparing mastectomy is one of the most commonly used techniques. This requires meticulous surgical expertise that preserves skin viability with complete removal of breast parenchyma. The best outcomes are expected with thin skin flaps that reduce the risk for residual breast tissue left behind. The approach of removing pectoralis fascia also minimizes the risk for residual breast tissue and close/positive margins of resection. Studies on patients treated with skin sparing mastectomy yield no increased incidence of local recurrence with this technique compared to conventional mastectomy [12, 17]. 

The relapses seen after breast conserving therapy in DCIS have a 50-50 chance for being either of *in situ* or invasive histology. However, relapses after mastectomy are mostly invasive as observed in this study and other published reports [13, 16]. The incidence of local-regional relapse rate we observed is similar to other reports in the literature Table 2. Rashtain et al. [16] studied 80 DCIS patients treated by mastectomy and <10 mm surgical margin. With a median followup of 61 months, they reported 7.5% (6/80) rate of local recurrence. Further, it was observed that 5 of these 6 recurrences occurred among the 31 patients with margins ≤2 mm compared to 1/6 failures among the 49 patients with greater than 2 mm negative margin. The study by Chan et al. [14] included 193 patients with DCIS treated with mastectomy. They reported a recurrence rate of 1.7%. Among this study cohort, 59 patients were identified to have <5 mm clear margin or positive margin. One of these 59 patients experienced a recurrence. The study by Chan et al. [14] reported an overall recurrence rate of 1.7% among the 59 patients with a <5 mm or positive margin following mastectomy. One of 19 patients (5%) with margin <1 mm had local relapse. On further review by nuclear grade only, they reported 3.3% crude risk of local recurrence among 30 patients who had a high nuclear grade [14]. Godat et al. [13] retrospectively reviewed 87 cases of DCIS treated by mastectomy between 1995 and 2006. The study included patients with microinvasive DCIS and Paget's disease associated with DCIS. With a mean follow-up time of 4.5 years, they observed a relapse rate of 1.1%. Carlson et al. [12] completed a retrospective review of 223 patients who underwent skin sparing mastectomy for DCIS. With a followup of 82.3 months, a relapse rate of 5.1% (*n* = 11) was reported. Among this group, 19 patients were identified with DCIS <1 mm from the surgical margin. Two of the 19 patients presented with local relapse. On univariate analysis, they showed that high tumor grade significantly influenced local relapse but surgical margin status did not reach statistical significance.

One of the 2 local relapses we observed was in a patient who 5 years subsequent to the initial diagnosis and treatment was diagnosed to be a mutation carrier. The retrospective design of the study precluded any systematic evaluation of risk for relapse based on presence of absence of BRCA mutations. Further, both the relapses observed were among the group of 24 patients that had close/positive margins. Although the association of margin status and relapse was statistically significant, we acknowledge that this observation is based on a relatively small sample size with short followup. In the literature, the final margin status is often associated with risk of local relapse [14, 15, 18, 19]. This probably represents an incomplete excision of the initial tumor at the time of mastectomy suggesting undetected residual burden left behind. Alternatively, it could be a new cancer in residual breast tissue.  Table 3 is a summary of published literature with a focus on the impact of unfavorable mastectomy margin. The average risk of relapse on the compiled data in Table 3 is 6.4% and is higher than the baseline risk of 0.9–4% observed for all patients (Table 2). Remarkably, the absolute numbers of relapses among patients with close/positive margins who are at a higher risk for relapses remain in single digit range. Based on these observations, one may conclude that not all positive/close margins will recur; however, most recurrences observed will have a close/positive margin. 

Kelley et al. [15] examined the use of the USC/Van Nuys Prognostic Index (USC/VNPI), a histopathologic scoring system for DCIS patients that takes into account tumor size, nuclear grade, necrosis, margin width, and patient age. They observed that mastectomy patients with a high USC/VNPI score may be at increased risk for local recurrence reporting overall local relapse rate of 3% at a mean follow-up time of 83 months. The probability of recurrence among patients scored between 10–12 was 9.6% compared to 0% for those scoring 4–9 at 12 years. The authors suggested that utilization of a clear margin on segmental excision serves as a surrogate for extensive DCIS. In this series, the average tumor extent of the 11 patients experiencing recurrence is 5.6 cm and all had multifocal disease. Although the authors report a correlation between their calculated USC/VNPI scores and recurrence, it is worth noting that the margin data used in the scores referred to premastectomy margins, that is, margins that were obtained after excision or biopsy but before mastectomy. The investigators might have arrived at different conclusions had the USC/Van Nuys Prognostic Index used the final mastectomy margins. More likely it is the final margin status of the mastectomy sample that would impact local recurrence [15]. 

Salvage after relapse has been reported by Kim et al. [18] This study reported on the largest number of postmastectomy recurrences to date and included a review of 10 chest wall relapses. Among these 10 patients, the common risk factors included young age, as well as multiquadrant DCIS, suggesting probability of residual breast tissue left behind after mastectomy. The patients in this study had their primary cancers treated at different institutions; therefore, this study cited a number of limitations, including lack of a comparison group, absence of data on mastectomy margins, and differences in standards of treatment among different institutions. Among the 10 recurrences observed, 9 were successfully salvaged with excision and radiation therapy at the time of relapse. The high salvage rate with surgery and radiation therapy suggests that delayed approach to PMRT for patients thought to have higher than baseline risk may be reasonable. With this approach, the routine application of PMRT with its attended late side effects can be avoided. 

In summary, the risk of relapse is low and there is evidence that salvage therapy including surgery and RT is highly effective [20, 21]. The rare local relapse after mastectomy limits our ability to acquire substantial evidence on the risk factors for relapse in DCIS. The low statistical power, to some extent, explains the lack of a clear consensus on this subject. Postmastectomy studies on DCIS that have tried to evaluate the impact of margin status have drawn conclusions based on very small numbers and through retrospective studies with inherent biases. Therefore, in clinical practice, the application of PMRT remains limited to an individualized risk determined at the discretion of the treating physicians. Such individualized assessment should include surgical technique used, extent of margin involvement, DCIS grade, and extent of tumor. Future research exploring the risk of relapse based on presence or absence of BRCA mutations and potential contribution of molecular breast cancer biomarkers including Oncotype Dx, HOXB-13, and others yet to be defined may guide the individual risk categorization for local relapse and accordingly the role for adjuvant therapies. 

## Figures and Tables

**Table 1:** Patient characteristics.

Clinical factor	Distribution (*n* = 211)
Age at diagnosis (years)	
(i) <40	20 (9.5%)
(ii) 41–50	86 (40.8%)
(iii) >50	105 (49.7%)
Race	
(i) White	123 (58.3%)
(ii) Black	30 (14.2%)
(iii) Others	58 (27.5%)
Primary method of diagnosis	
(i) Breast Exam	48 (22.7%)
(ii) Mammogram	151 (71.5%)
(iii) Other imaging	12 (5.7 %)
Nuclear Grade	
(i) Grade 1	22 (10.4%)
(ii) Grade 2	97 (45.9%)
(iii) Grade 3	92 (43.6%)
Final margins	
(i) Negative	187 (88.6%)
(ii) Close (<1 mm)/Positive	19 (9%)/5 (2.4%)
Receptor status	
(i) ER Positive	42 (19.9%)
(ii) ER Negative	107 (50.7%)
(iii) Unknown	62 (29.4% )

**Table 2:** Local recurrence in DCIS following mastectomy—Summary of literature.

Author (year)	No: of	% LR	Length of follow up
	patients	recurrence	In years (median)
Silverstein (1990) [6]	167	2%	10 years
Ciatto (1990) [8]	210	3%	5.5 years (mean)
Cataliotti (1992) [9]	103	3%	10.6 years
Warneke (1995) [10]	75	1.3%	3.6 years
Ringberg (2000) [11]	119	4%	5 years
Carlson (2007) [12]	223	3.1%	6.8 years
Godat (2009) [13]	83	1.1%	4.5 years
Chan (2010) [14]	193	1.7%	8 years
Kelley (2011) [15]	496	3%	6.9 years
Present study	211	0.9%	4.6 years

**Table 3:** Local-regional relapse following an unfavorable surgical resection margin.

Author	Close	No: of	No: of	Follow up
	margin/positive	patients	relapses	in months
Godat [13]	<5 mm	39	1	54 months
Rashtian [16]	<2 mm	31	5	61 months
Chan [14]	<5 mm	59	1	96 months
Carlson [12]	<1 mm	19	2	82.3 months
Present study	<1 mm	24	2	55 months

TOTAL		172	11 (6.4%)	
